# Rbt1 Protein Domains Analysis in *Candida albicans* Brings Insights into Hyphal Surface Modifications and Rbt1 Potential Role during Adhesion and Biofilm Formation

**DOI:** 10.1371/journal.pone.0082395

**Published:** 2013-12-05

**Authors:** Céline Monniot, Anita Boisramé, Grégory Da Costa, Muriel Chauvel, Marc Sautour, Marie-Elisabeth Bougnoux, Marie-Noëlle Bellon-Fontaine, Frédéric Dalle, Christophe d’Enfert, Mathias L. Richard

**Affiliations:** 1 INRA, UMR1319 Micalis, Jouy-en-Josas, France; 2 AgroParisTech, UMR Micalis, Thiverval Grignon, France; 3 Institut Pasteur, Unité Biologie et Pathogénicité Fongiques, Département Génomes et Génétique, Paris, France; 4 INRA, USC2019, Paris, France; 5 UMR Agroécologie 1347 Agrosup/INRA/Université de Bourgogne, Laboratoire Microbiologie Environnementale et Risque Sanitaire (M.E.R.S.), Dijon, France; 6 Université Paris Descartes, Sorbonne Paris Cité, Paris, France; 7 Assistance Publique – Hôpitaux de Paris Unité de Parasitologie-Mycologie, Service de Microbiologie, Hôpital Necker-Enfants-Malades, Paris, France; Institute of Biology Valrose, France

## Abstract

Cell wall proteins are central to the virulence of *Candida albicans*. Hwp1, Hwp2 and Rbt1 form a family of hypha-associated cell surface proteins. Hwp1 and Hwp2 have been involved in adhesion and other virulence traits but Rbt1 is still poorly characterized. To assess the role of Rbt1 in the interaction of *C. albicans* with biotic and abiotic surfaces independently of its morphological state, heterologous expression and promoter swap strategies were applied. The N-terminal domain with features typical of the Flo11 superfamily was found to be essential for adhesiveness to polystyrene through an increase in cell surface hydrophobicity. A 42 amino acid-long domain localized in the central part of the protein was shown to enhance the aggregation function. We demonstrated that a VTTGVVVVT motif within the 42 amino acid domain displayed a high β-aggregation potential and was responsible for cell-to-cell interactions by promoting the aggregation of hyphae. Finally, we showed through constitutive expression that while Rbt1 was directly accessible to antibodies in hyphae, it was not so in yeast. Similar results were obtained for another cell wall protein, namely Iff8, and suggested that modification of the cell wall structure between yeast and hyphae can regulate the extracellular accessibility of cell wall proteins independently of gene regulation.

## Introduction

*Candida albicans* is a major opportunistic fungal pathogen [1]. It can cause both superficial mucosal infections and life-threatening systemic infections in healthy and immunocompromised individuals, respectively [2]. In the latter case mortality rates can reach 50%. *C. albicans* pathogenicity is a multi factorial process: the main characteristics of *C. albicans* are its abilities to switch between yeast and filamentous (hyphal) growth modes [3], to adhere to various substrates and to resist the immune system. It is generally accepted that hyphae represent the invasive morphological form as shown by histological images of *C. albicans* invading kidney [4]. However, yeast cells can also be found in infected organs and it appears that this morphology is important for dissemination via the bloodstream [5]. Dimorphism is controlled by transcriptional factors such as Efg1 and Cph1 [6] or by repressors such as Nrg1 and Tup1 [7-9]. Morphology is not the only trait governed by these regulators: expression of several morphology-associated genes is also controlled by these factors. Indeed, hypha formation is concomitant to the synthesis and cell surface exposure of hypha-specific adhesins. These adhesins mediate the attachment of cells to plastic surfaces or host cells but also the adherence of cells to one another and thereby contribute to biofilm formation [10]. The ability to form biofilms on surfaces in the host or on implanted medical devices enhances *C. albicans* virulence by allowing the colonization of various niches and by providing reservoirs for infection and conditions favoring resistance to antimicrobial drugs.

The well-described hypha-specific adhesins Als3 and Hwp1 are members of two distinct families of glycosylphosphatidylinositol (GPI)-anchored proteins. Als3 belongs to the ALS (Agglutinin Like Sequence) family and was shown to play, along with Als1 and Als5, a crucial role in many different pathogenic processes such as adhesion to epithelial and endothelial cells, promotion of clathrin-mediated endocytosis of hyphae, biofilm formation and iron acquisition [11]. Expression of *ALS3* in a *Saccharomyces cerevisiae* surface display system allowed attachment of the recombinant strain to epithelial cells, endothelial cells and extracellular matrix proteins [12] as well as to polystyrene [13]. More recently, Ramsook et al. [14] identified sequences with a high β-aggregation potential in Als5. They showed that this sequence in the threonine-rich region of Als5 proteins mediates amyloid formation, and that amyloid binding dyes can inhibit the cell aggregation in the S. *cerevisiae* surface display model [14]. Another study by the same laboratory showed that a single substitution in the amyloid sequence was sufficient to disrupt aggregation in the S. *cerevisiae* display model, but more importantly they showed the role of these sequences using inducing and inhibiting peptides in *C. albicans* live cells [15]. Additionally, atomic force microscopy (AFM) was used to illustrate that these amyloid sequences were responsible for the clustering of the adhesins on the cell surface, a phenomenon that might have a crucial role for *C. albicans* adhesion properties [15,16].

The other well-characterized adhesin Hwp1 is a member of a three protein family with Hwp2/Pga8 and Rbt1 (namely Family 12 of the 23 families of GPI-anchored proteins identified in the *C. albicans* genome [17]). This family is conserved to some extent in *C. dubliniensis*, *C. tropicalis* (no Hwp orthologue) and *C. parapsilosis* (no Hwp orthologue) but absent from other fungi [17]. Hwp1 was originally described as being required for the covalent attachment of *C. albicans* to host epithelial cells, following host transglutaminases activity on the Hwp1 N-terminal domain [18]. Further studies have portrayed Hwp1 involvement in biofilm formation and adhesion to plastic [13,19,20] and shown Hwp2/Pga8 contribution to tolerance to oxidative stress, invasive growth, adhesion and biofilm formation [21,22]. In contrast, little is known about Rbt1. Although this protein has been predicted to be GPI-anchored [23], its precise localization is unknown. Braun et al. [7] initially characterized Rbt1 as a protein Repressed By Tup1 along with Hwp1. Furthermore, they showed in a rabbit cornea model and a mouse systemic infection model that the rbt1-/- strain had a significantly reduced virulence, but no restoration of the wild type phenotype was observed after *RBT1* re-integration. Studying its role in mating and biofilm formation showed that rbt1-/- mutants had a mild defect in biofilm formation but that Rbt1 did not seem to play, in *C. albicans*, a role equivalent to that of agglutinin in *S. cerevisiae* [19]. Notably, Hwp1, Hwp2 and Rbt1 share similarities mostly in their C-terminal domain, suggesting that the N-terminal domains of Hwp2 and Rbt1 might have substrate specificity other than that elicited by the Hwp1 N-terminal domain. In this respect, similarity of the Rbt1 N-terminal domain with the N-terminus of *S. cerevisiae* Flo11 suggested that the adhesiveness of Rbt1 was mediated by this part of the protein. Flo11 is a member of the flocculin family in *S. cerevisiae*, where it is required for diploid pseudohyphal formation and haploid invasive growth [24,25] but the mechanism of cellular adhesion mediated by Flo11 is not well understood. Goossens and Willaert [26] showed that the N-terminal domain of Flo11 that does not contain the mannose-binding domain PA14 present in the other flocculins (Flo1, Flo5, Flo9 and Flo10) was unable to bind mannose. They suggested that the ability of this domain to self-interact might explain the cell-to-cell interaction capacity of *FLO11*-expressing cells. Indeed, Flo11 involvement in cell-surface adhesion during invasive growth as well as in cell-to-cell interaction during biofilm formation has been previously reported [27,28]. 

Adhesion of *C. albicans* to epithelial cells has been also associated with modifications of cell surface hydrophobicity [29]. Based on cell surface ultrastructures and biochemical analyses Hazen & Hazen [30] have proposed that *C. albicans* hydrophobicity was not determined by differences in surface hydrophobic proteins but by the presence of hydrophilic surface fibrils. They showed that alteration of the *C. albicans* cell wall fibrillar outer layer resulted in exposure of cryptic hydrophobic proteins. In this paper, we integrated the two models with a study of the protein function as well as the biochemical properties of the cell surface to present a structure-function analysis of Rbt1 focused on its cellular localization and its adhesion properties. Our results have identified two key domains of Rbt1 involved in adhesion to abiotic and biotic surfaces and shown that Rbt1 cell surface exposure is influenced by the differing yeast and hypha cell wall structures. Thus, our data reveal a new type of regulation of protein exposure at the surface independent of gene regulation or post-translational modification.

## Materials and Methods

### Strains

The *Saccharomyces cerevisiae* BY4742 strain [31] was used for heterologous expression of *Candida albicans* Rbt1 protein. The *C. albicans* strain used for DNA amplification was BWP17 [32], constitutive expression of *RBT1* was performed in a DAY286 [33] context and CAI-4 [34] was used for expression of the tagged copies of Rbt1-V5 (see Table 1). BY4742 and its derivatives were grown at 28°C either in liquid YPD (1% glucose, 1% bacto peptone, 1% yeast extract) or in liquid YNB N_5000_ (1.7% yeast nitrogen base without ammonium sulphate and amino acid from Difco + 1% glucose + 0.5% ammonium sulphate) supplemented with 10 mg.mL^-1^ lysine, 10 mg.mL^-1^ leucine and 10 mg.mL^-1^ histidine. *C. albicans* overexpressing strains and recombinant CAI-4 strains were cultivated at 30°C in liquid synthetic medium (0.67% yeast nitrogen base with ammonium sulphate and without amino acid from Difco, 2% glucose and 0.17% amino acid drop out mix) supplemented with 10 mg.mL^-1^ uridine. For biofilm formation in micro-fermenter, liquid YNB N_5000_ was supplemented with 20 mg.mL^-1^ arginine, 20 mg.mL^-1^ histidine, 20 mg.mL^-1^ uridine and 200 mg.mL^-1^ methionine (GHAUM medium; [35]). For filamentation induction, cultures were switched to liquid synthetic medium buffered at pH7 with 100 mM Hepes and incubated at 37°C. For the aggregation studies, the filamentation was induced as described above and were cultured 24h at 37°C before observation. In the case of the addition of the peptides: the peptide was added when the cells were transferred to the pH7 buffered medium (VTTGVVVVT at 2 μg.mL^-1^ and V5N at 20 μg.mL^-1^); the growth at 37°C was only of 2h and the 24h left were at room temperature on the bench to keep loose aggregates, and differences visible. A *rbt1-/-* mutant strain and its wild type control from Ene and collaborators were included in this study (Table 1) [19].

**Table 1:** List of strains used in this study.

**Strains**	**Parental strains**	**Genotypes**	**References**
BY4742		*MATa his3*Δ*1 leu2*Δ*0 lys2*Δ*0 ura3*Δ*0*	[31]
CAI-4	SC5314	*ura3*Δ*::imm434*/*ura3*Δ*::imm434*	[34]
BWP17	CAI-4	*ura3*Δ::*imm434*/*ura3*Δ::*imm434;his1*Δ*::hisG/his1*Δ*::hisG; arg4*Δ*::hisG/arg4*Δ*::hisG*	[32]
DAY286	BWP17	*ura3*Δ::*imm434*/*ura3*Δ::*imm434; his1*Δ::*hisG*/*his1*Δ::*hisG; ARG4*::*URA3*::*arg4*Δ::*hisG*/*arg4*Δ::*hisG*	[40]
DAY185	BWP17	*ura3*Δ:: *imm434/ura3*Δ*::imm434; HIS1::his1*Δ*::hisG/his1*Δ*::hisG; ARG4::URA3::arg4*Δ*::hisG/arg4*Δ*::hisG*	[40]
RBY1175	RBY1118	*arg4/arg4*	[19]
CAY171	RBY1132	*leu2/leu2 his1/his1 arg4/arg4 rbt1*Δ::*LEU2/rbt1*Δ::*HIS1*	[19]
VIF105	CAI-4	Same as CAI-4 but RPS10/rps10ΔpExpV5-IFF8	[36]
VIF106	CAI-4	Same as CAI-4 but RPS10/rps10ΔpExpV5-DCW1	[36]
VIF201	BY4742	Same as BY4742 + plasmid pBC542(*URA3*+*RBT1SL*)	This study
VIF202	BY4742	Same as BY4742 + plasmid pBC542(*URA3*+*RBT1FL*)	This study
VIF203	BY4742	Same as BY4742 + plasmid pBC542(*URA3*+ΔN*RBT1SL*)	This study
VIF204	BY4742	Same as BY4742 + plasmid pBC542(*URA3*+ΔN*RBT1FL*)	This study
VIF205	BY4742	Same as BY4742 + plasmid pBC542(*URA3*)	This study
VIF206	BY4742	Same as BY4742 + plasmid pBC542(URA3+EAP1)	This study
VIF207	DAY286	Same as DAY286 but *pRBT1SL::HIS1-pTEF1-RBT1SL*	This study
VIF208	DAY286	Same as DAY286 but *pRBT1FL::HIS1-pTEF1-RBT1FL*	This study
VIF209	CAI-4	Same as CAI-4 but *RBT1::RBT1SL-V5-URA3*	This study
VIF210	CAI-4	Same as CAI-4 but *RBT1::RBT1FL-V5-URA3*	This study
VIF211	CAI-4	Same as CAI-4 but *RPS1::pACT-RBT1SL-V5-URA3*	This study
VIF212	CAI-4	Same as CAI-4 but *RPS1::pACT-RBT1FL-V5-URA3*	This study

### Construction of epitope-tagged copies of Rbt1 proteins

To allow detection of the Rbt1 protein, a V5-tagged version was constructed in which the V5 epitope was inserted in the Rbt1 sequence between amino acid 273 and 274. This site of insertion was chosen because a Kex2 site is predicted in the N-terminal domain of the protein and the sequence at position 273 was part of no conserved domain. For this purpose, two couples of primers were designed (primers 12 to 15, see Table 2) and used separately in a first amplification. Since RBT1V5F820 and RBT1V5R819 contained 18 complementary bases, the two PCR products were mixed, denatured and the temperature was slowly decreased to allow cross-hybridization between the simple strands of the two amplicons. A second amplification was then performed using this reaction as template and the two external primers (RBT1ATGHind and Rbt1STOPPst). The product was cloned in pGEMT-Easy (Promega), sequenced and then subcloned in the pExp-V5 expression vector [36] at the *Hin*dIII and *Nsi*I unique restriction sites. Each of the two *RBT1* alleles was cloned and the recombinant vectors were inserted in CAI-4 either at the *RPS1* locus after linearization by *Stu*I or at the *RBT1* locus after linearization by *Afl*II that cuts in the *RBT1* coding sequence. Correct integrations were checked by PCR using respectively the RP10-1250R and PACTGLUC-4652 (Table 2, 16-17) or the PRBT1F and V5R (Table 2, 18-19) couples of primers. While integration at *RPS1* promoted expression of tagged *RBT1* alleles from the *ACT1* promoter (VIF211 and 212), integration at *RBT1* promoted expression of these alleles from their native promoter (VIF209 and 210, see Table 1).

**Table 2:** List of primers used in this study.

**Number**	**Name**	**Sequence**
*Construction of the pBC542 recombinant vectors expressing entire proteins*		
1	RBT1AttB1ATG	GGGACAAGTTTGTACAAAAAAGCAGGCTCAACTATGAGATTTGCAACTGC
2	RBT1AttB22098R	GGGACCACTTTGTACAAGAAAGCTGGGTACTTCGAATGAAGAGACTGAAGC
3	EAP1AttB1ATG	GGGACAAGTTTGTACAAAAAAGCAGGCTAAAATGAAAGTTTCTCAAATTTTACC
4	EAP1AttB21894R	GGGACCACTTTGTACAAGAAAGCTGGGTACTTCAAAAGTGGAAACTTGAGC
*Construction of the pBC542 recombinant vectors expressing truncated proteins*		
5	RBT1XbaATG	CCC**TCTAGA**CTATGAGATTTGCAACTGCC
6	RBT1Nhe60R	CCC**GCTAGC**CTCAGTGGATAAAATGTAG
7	RBT1Xba805	CCC**TCTAGA**GACTGTCAATGTGACCCC
8	RBT1Xho2090R	CCC**CTCGAG**GAAGAGACTGAAGCAATAGTG
*Exchange of the RBT1 promoter for the TEF1 promoter*		
9	JRBT1PUp	CTTAATATCTACAAAGATAGCCTGCTGTAATGACAGTATTTTCTTTTAATTGCTCATGTCATTTAG TATTTACGAAAATGAGTCTGGACGATACATCGATTTCCCAGTCACGACGTTC
10	JRBT1PDo	GTGGAATACAATTAAAGATGTCACCCAATAATGGGAAAGTAGCCTCAGTGGATAAAATGTAGTA AGCGAGGGCAGCGAGTTGGGCAGTTGCAAATCTCATGATTGATTATGACTATAATGTG
11	PTEFMluF	ACGCGTGTAAACGCTGATACGGCAT
*Construction of RBT1 V5-tagged copy*		
12	RBT1ATGHind	GCAAGCTTCAACTATGAGATTTGCAACTGCCC
13	RBT1V5F820	CCAAATCCATTGTTGGGTTTGGATTCAACTACCCCATCTCCATCAACTACC
14	RBT1V5R819	ACCCAACAATGGATTTGGAATTGGTTTACCGTCACATTGACAGTCCCAAC
15	Rbt1STOPPst	CCCTGCAGCAAGACCAATAATAGC
16	RP10-1250R	CGTATTCACTTAATCCCACACT
17	PGLUC-4652	GTTTTGTACCTATATGACTCTTC
18	PRBT1F	AAATCTCGTATTAGTCATTCGC
19	V5R	CCAAACCCAACAATGGATTTGG
*RT-Qpcr*		
20	RBT1qFb	TCAATGCCGCATTTGTCGTGTCT
21	RBT1qRb	AAGGCCAGGTTCAATTGGACAG
22	pACT1R	ACAGAGTATTTTCTTTCTGGTGGAGCA
23	pACT1F	AGTGTGACATGGATGTTAGAAAAGAATTATACGG

### Cell lysis and preparation of cell walls and soluble protein fractions

*C. albicans* CAI-4 transformed cells were harvested by centrifugation, washed with 10 mM Tris-HCl containing 10 mM NaN_3_ and disrupted in ice after resuspension in lysis buffer (50 mM Tris-HCl pH7.5, 150 mM NaCl) containing protease inhibitors (complete, EDTA-free from Roche) and glass beads in a Bead-Beater 24™ (MP Biomedicals, California, USA) four times for 20 seconds each with 5 minutes in ice between each round. Subsequently, the lysate was collected and further centrifuged at 1,000 x *g* for 10 minutes at 4°C to collect a low-speed supernatant (S_1000_) and a low-speed pellet (C_1000_). To obtain a plasma membrane-enriched fraction, the low-speed supernatant was further centrifuged at 100,000 x *g* for 1 hour at 4°C and the supernatant corresponding to the soluble protein fraction was removed. For membrane protein solubilisation, the high-speed pellet was resuspended in 50 mM Tris-HCl pH7.4 containing 2% SDS and anti-proteases and then heated for 5 minutes at 95°C. After 5 minutes of centrifugation at 10,000 x *g*, the non-solubilized material was discarded. For cell wall protein extraction, the low-speed pellet was washed extensively with 1M NaCl and the resulting cell walls were boiled twice in the presence of 50 mM Tris-HCl pH8, 100 mM EDTA pH8, 2% SDS to solubilize the non-covalently linked cell wall proteins and to remove any contaminant derived from the cytosol and/or plasma membrane. SDS-extracted cell walls were then extensively washed with H_2_O, resuspended in 20 mM NaAc pH4.5 and incubated for 3 hours at 37°C with purified recombinant β-1,6-glucanase [37]. After 5 minutes of centrifugation at 10,000 x *g*, the insoluble material was discarded and the solubilized proteins were concentrated on Microcon™ 50 (Millipore) before Western blot analysis.

### Western blot analysis

Extracted proteins were separated by SDS-polyacrylamide gel electrophoresis on NuPAGE^R^Novex Tris-acetate 3-8% pre-cast gels (Invitrogen) in NuPAGE^R^Novex Tris-acetate Running buffer (Invitrogen) using the XCell Mini-Cell system from Invitrogen. The proteins were transferred onto a nitrocellulose membrane (PROTRAN) for Western blot analysis. Following transfer, membranes were rinsed in Phosphate-Buffered Saline (PBS) and blocked in PBST (PBS plus 0.1% Tween 20) + 2% skimmed milk from Difco for one hour at room temperature. The membrane was then incubated overnight at 4°C in PBST containing a 1:5000 dilution of the monoclonal anti-V5 antibody (Invitrogen). After 3 washes in PBST, one-hour incubation in the presence of peroxidase-conjugated anti-mouse IgG antibodies (GE Healthcare) was performed. The membranes were washed three times before detection of the signal using the Enhanced Chemi Luminescence (ECL)^+^ detection system (GE Healthcare).

### Immunofluorescence detection

*S. cerevisiae* and *C. albicans* cells were fixed in 4.6% formaldehyde for 40 minutes and after washes were placed on polylysine coated slides, then blocked in PBS + 0.5% BSA and incubated successively in the presence of: (i) monoclonal anti-V5 antibodies (Invitrogen) for *C. albicans* cells and anti-mouse IgG-Cy3 conjugated antibodies (SIGMA) in PBS + 0.5% BSA for 1 hour in the dark; or (ii) polyclonal anti-HA antibodies (MP Biomedicals) for *S. cerevisiae* cells and anti-rabbit IgG-Cy3 conjugated antibodies (SIGMA) in PBS + 0.5% BSA for 1 hour in the dark. After washes in PBS, DAPI was added (2 µg.mL^-1^ final concentration) and rinsed before the mounting step. For permeabilization, cells grown to OD=1-2 were washed and resuspended in phosphate buffer pH 7.5 with a 5X concentration before being fixed in 8-fold diluted 37% formaldehyde and incubation for 40 minutes at room temperature. After 3 washes in phosphate buffer, cells were incubated for 20 minutes at 37°C in Sorbitol buffer + Zymolyase 100T (0.5 mg.mL^-1^ final). 2 volumes of cold sorbitol buffer were added before centrifugation and 2 washes in sorbitol buffer. Cells were resuspended in sorbitol buffer before proceeding with immunofluorescence. Cells were examined by fluorescence microscopy (Olympus BX51) with 512-nm excitation and 565-nm emission filters using an Olympus 100X oil immersion objective and 10X oculars.

### Flow Cytometry (FCM) analysis

FCM analyses were performed with a CyFlow SL cytometer (PARTEC, Sainte-Geneviève des Bois, France). The FCM analyses were performed using logarithmic gains and specific detector settings, adjusted on a sample of unstained cells, to eliminate cellular auto-fluorescence. Data were collected and analyzed with FlowMax software (PARTEC). The samples were prepared as follows: cells were fixed in 4.6% formaldehyde for 40 minutes and after two washes in PBS were incubated successively in PBS + 0.5% BSA + polyclonal anti-HA antibodies (MP Biomedicals) for 1h in the dark and in PBS + 0.5% BSA + polyclonal anti-Rabbit-IgG-fluorescein antibodies (Jackson Immuno Research) for 30min in the dark. After a final wash in PBS the cells were resuspended in water for FCM analysis. The experiment was repeated 3 times. 

### Cloning in *Saccharomyces cerevisiae* expression vectors

To express the *C. albicans* Rbt1 protein at the S. *cerevisiae* cell surface, fragments corresponding to the two allelic coding sequences were amplified from BWP17 genomic DNA using primers 1 and 2 (Table 2) and cloned in the pBC542 vector [38] using the Gateway recombinase-based cloning system (Invitrogen). The *EAP1* sequence, amplified using primers 3 and 4 (Table 2), was also cloned in this vector as a positive control of adherence. To express Rbt1 variants lacking the N-terminal domain, two different fragments were produced: (i) the Rbt1 signal peptide using primers 5 and 6; (ii) and the Rbt1 C-terminal sequences from amino acid 273 using primers 7 and 8. After restriction with *Nhe*I and *Xba*I and further ligation of the two fragments, the final products were digested with *Xba*I and *Xho*I and cloned in the pBC542 vector at the *Xba*I and *Xho*I unique sites. The recombinant plasmids were sequenced and transferred into BY4742 as well as pBC542 that was used as a negative control. BY4742 Ura^+^ recombinant strains were selected on minimal medium supplemented for lysine, histidine and leucine.

### Cell surface hydrophobicity

Cell surface hydrophobicity was determined by the previously described MATS method (for Microbial Adhesion to Solvents) with some modifications [39]. Cells grown overnight in liquid YNB N_5000_ medium supplemented with leucine, histidine and lysine at 28°C, were washed twice in physiological water and resuspended at an OD_600_=0.8. 150 µl of decane was added to 1.5 ml of cell suspension. The samples were shaken 15 seconds in the hand and then vortexed 90 seconds. After 15 minutes at room temperature, the OD_400_ of the aqueous phase A_1_ was measured. The OD_400_ of the sample without decan corresponded to A_0_. To calculate the percentage of cell surface hydrophobicity (CSH), we used the formula: CSH = (1 – A_1_/A_0_) x 100.

### Construction of *RBT1* overexpressing strains

To exchange the *RBT1* native promoter for the *C. albicans TEF1* promoter, primers JRBT1PUp and JRBT1PDo (see Table 2, 9-10) and pHISOx, a pGEMT-Easy derivative harboring the *C. albicans HIS1* gene (promoter and terminator included) cloned 80 bp upstream to the *TEF1* promoter were used to generate a PCR product of 2.5 kbp containing successively: a 100 nucleotide-long sequence at the 5’ end identical to the *RBT1* 5’ upstream sequence located at position -1567 from the *RBT1* start codon, the *C. albicans HIS1* gene, a 412 nucleotide-long sequence corresponding to the *C. albicans TEF1* promoter and at its 3’ end 100 nucleotides of the *RBT1* coding sequence from the start codon. The PCR product was purified, concentrated and used for transformation of the *C. albicans* DAY286 strain, subsequent selection was made using the histidine auxotrophy. Correct promoter replacement after a double recombination event between the native promoter and the *RBT1* promoter flanking regions of the amplicon was verified by PCR on the genomic DNA using a *TEF1* forward primer internal to the cloned sequence: PTEFMluF (Table 2, 11) and a *RBT1* reverse primer complementary to a portion of the coding sequence: RBT1Xho2290R (Table 2, 8). Two strains, OEx*RBT1SL* and OEx*RBT1FL* (VIF 207 and 208, Table 1), corresponding respectively to the expression of a short *RBT1* allele and a long *RBT1* allele under the control of the *TEF1* promoter were obtained. 

### RT-qPCR analysis

To measure *RBT1* expression in the two overexpressing strains, we performed RT-qPCR in yeast and in hypha forms. Yeast cells were grown overnight at 30°C in SC pH7, diluted at OD_600_=0.25 and further grown in the same conditions to OD_600_=1. To obtain hyphae, cells were grown overnight at 30°C in SC pH5, diluted in SC pH7 at OD_600_=1 and then further incubated for 2 hours at 37°C. The yeast and hypha forms were treated with the same protocol described below. The cultures were centrifuged and the pellets were frozen in liquid nitrogen and stored at -80°C. Total RNA was extracted following the instructions of the RNeasy Mini QIAGEN kit. To eliminate genomic DNA contamination, total RNA was treated with RNase-free DNase I (DNase Ambion). RNA was measured using the Nanodrop (ND-1000, ThermoScientific) to evaluate the concentration and the quality with A260/280 and A260/230 ratios. mRNAs were reverse transcribed using the SuperScript™ III Reverse Transcriptase Kit (Invitrogen) according to the manufacturer’s instructions. Expression levels of *RBT1* in the two cell forms were determined using primers RBT1qFb and RBT1qRb (Table 2, 20-21) in the two overexpressing strains and their control DAY185 [40] and compared to *ACT1* expression levels (Table 2, 22-23).

### Adhesion to polystyrene and biofilm formation

*Saccharomyces cerevisiae* transformed cells were grown overnight at 28°C in liquid YNB N_5000_ medium supplemented with leucine, histidine and lysine. Cells were adjusted in fresh medium to OD_600_=1. 500 µl of cell suspension were allowed to adhere for 60 minutes at 28°C to wells of sterile 24-wells polystyrene plates treated for tissue culture (BD Falcon). After 60 minutes, non adherent cells were removed by washing three times with 1 ml of PBS. For biofilm formation, 500 µl of fresh supplemented YNB N_5000_ medium was added and plates were further incubated for 24 hours at 28°C without agitation. Adherence and biofilm formation were assessed by cells staining with 500 µl of 0.5% crystal violet per well during 10 min at room temperature. After two washes with 1 ml of H_2_O, the biomass was quantified after addition of 500 µl of 10% acetic acid per well and measurement of the OD_595_ of the solution after 10 minutes of incubation. In case of very high level of crystal violet absorbance, serial dilutions were performed to keep the OD_595_ in a 0.1-5 range. 

*C. albicans* biofilm formation was assessed using a micro-fermenter system and Thermanox™ (Nunc) as substrate [35]. Inocula were prepared from *C. albicans* early stationary-phase cultures grown in GHAUM medium at 30°C in an orbital shaker and diluted to OD_600_=1. Biofilms were produced in micro-fermenters that consist of a glass vessel with a 40-ml incubation chamber where two glass tubes are inserted to drive the entry of GHAUM medium and air. Used medium is evacuated through a third tube. Medium flow is controlled by a recirculation pump (Ismatec) and pushed by the pressured air. Plastic slide (Thermanox™) glued to a glass spatula was immersed in the inoculum for 30 minutes at room temperature. After this adhesion period, the spatula was transferred to the chamber and incubated at 37°C for 40 hours with the medium flow set to 0.6 ml/min and air supplied at 10^5^ Pa.

### Adherence to HeLa and Caco-2 cells

For adherence to HeLa cells (from ECACC ref. 93021013), *C. albicans* cells were grown to OD_600_=0.8 in minimal SC medium buffered at pH7 at 30°C. Cells were resuspended at a concentration of 10^4^ cells/ml in H10 (Supplemented DMEM Gluta Max from Gibco + 1% of non-essential amino acids + 1% of Penicillin and Streptomycin + 10% of fœtal bovin serum). 1 ml/well of the cell suspension was added to confluent HeLa cell monolayer in 6-wells culture cell plates (BD Falcon) and plates were incubated for 45 minutes at 37°C. After this incubation time, half of the plate (3 wells) was washed three times with 5 ml of warm PBS. These wells constituted the assay and the unwashed wells represented the inoculum. HeLa and yeast cells of each well were then scraped in 1 ml of PBS and collected in 1.5 ml Eppendorf tube. Cells were diluted at 1/10 for the inoculum and at 1/5 for the assay in PBS and 100 μl were spread on solid YPD plates and colonies were counted after 48h of incubation at 30°C. Adhesion was quantified as the number of colonies on plates corresponding to the assay divided by the number of yeast cells initially added to the HeLa monolayer. 

For adherence to Caco-2 cells (from ATCC ref. HTB-27), assays were performed according to a previously described protocol [41]. Briefly, Caco-2 cells grown on 12 mm glass coverslips were inoculated with ~10^4^ log phase yeast cells of *C. albicans* overexpressing strains and control. After 30 minutes at 37°C, the cells were washed three times with PBS to remove non-adherent yeasts and cells were fixed with paraformaldehyde 3.6 % for 10 minutes. Adherent *C. albicans* cells were stained with calcofluor white and quantified by epifluorescence (Eclipse E600, Nikon) using a filter set to detect calcofluor white. The percentage of adhesion in each culture was determined as the ratio of the number of adherent *C. albicans* cells on the entire surface of the coverslip to the number of *C. albicans* cells inoculated. Each condition was tested in quadruplicate.

## Results

### Rbt1: a protein with multiple domains and sequence variability

**Figure 1:**
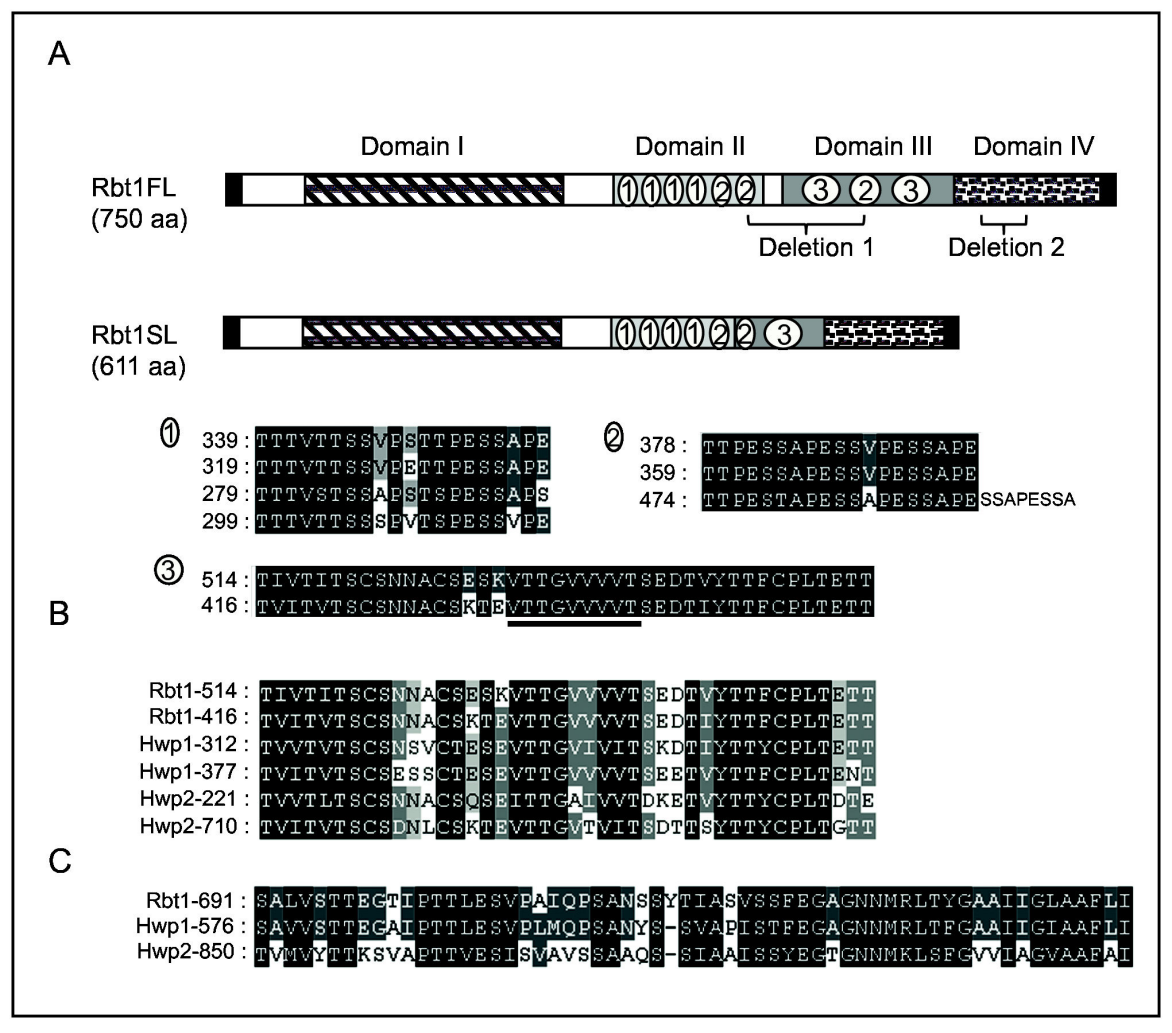
Rbt1 protein description and sequence similarities. A/ Schematic representation of the two Rbt1 proteins domain organization with for Rbt1FL from left to right: the signal peptide (black box, aa 1-21); the domain I matching the Flo11 superfamily (dashed box, aa 71-216); the domain II, a Ser/Thr-rich region containing the imperfect repeats 1 and 2 (light grey box, aa 279-396); the domain III (dark grey box, aa 416-555) containing the two 42 amino acid-long repeats (repeat 3) comprising the sequence with a high β-aggregation potential (underlined) and another repeat 2 (containing two additional repeats PESSA); the domain IV (dotted box, aa 556-729) which precedes the GPI anchor addition signal (black box, aa 729-750). The two deletions in the Rbt1SL protein are represented by: Deletion 1 (aa 378-487) and Deletion 2 (aa 612-640). B/ Sequence similarities within the Hwp1 family in the 42 amino acid-long repeat 3. C/ Sequence similarities within the Hwp1 family in the last 60 aa.

In order to perform a structure-function analysis of the Rbt1 protein, we amplified and sequenced the *RBT1* coding sequence from the *C. albicans* BWP17 strain (Table 1). On the basis of both similarity and amino acid (aa) composition of the 750 aa long sequence deduced from the first allele, we distinguished four domains, referred to as domain I, II, III and IV (Figure 1). The first 21 aa residues are predicted to act as a signal peptide targeting the Rbt1 protein to the secretory pathway. Following this sequence, domain I extends from aa 71 to 216, and matches the Flo11 superfamily of the NCBI Conserved Domain Database (CDD) (**pfam10182**) [42]. Domain II lies from aa 279 to 396 and displays 4 repetitions of a 20 amino acid-long motif containing more than 50% of serine and threonine residues (Figure 1A) followed by 2 sequences of 19 aa residues harboring 3 repetitions of a “PESS/TA/V” motif. Domain III extends from aa 416 to 555 and shows 2 copies (respectively from aa 416 to 457 and 514 to 555) of the conserved flocculin type 3 repeat (**pfam13928**) found close to the C-terminus of the S. *cerevisiae* flocculation protein Flo9 (Figure 1A). This latter motif is also found twice in Hwp1 as well as in Hwp2 but is absent in proteins of the Als family (Figure 1B). In Rbt1, in contrast to Hwp1, the two copies are more distant and the sequence that separates them contains 5 repetitions of the “PES/TSA/V” motif from aa 474 to 500 (Figure 1A). Domain IV ranging from amino acid 556 to the C-terminal GPI anchoring signal is mainly composed of alanine (19%), serine (18.4%) and proline (17.8%) residues. The omega site for GPI anchor addition is predicted to be a glycine at position 729 using the big-PI Fungal Predictor [43]. The last 60 amino acids display 80% identity with those in the Hwp1 C-terminus (Figure 1C) but less than 50% with those in the Hwp2 C-terminus. 

The second allelic sequence amplified from BWP17 was shorter and was predicted to be translated into a 611 amino acid-long protein. The two proteins, respectively called Rbt1FL (full length) and Rbt1SL (short length) differed in domains II, III and IV. Specifically, the shorter protein Rbt1SL displayed a 110 amino acid-long deletion (positions 377-488) overlapping domains II and III, therefore yielding a protein with only one copy of the flocculin type 3 repeat (see Figure 1A); and a 29 amino acid-long deletion (positions 611-641) in domain IV (Figure 1A). 

We used 80 *C. albicans* clinical isolates in order to assess the distribution of the *RBT1FL* and *RBT1SL* alleles. Interestingly, although several isolates contained alleles of different sizes, none of them harbored an allele of the size of *RBT1SL* except for SC5314. BWP17 and its parental strains are thus the only strains harboring the *RBT1FL/RBT1SL* allelic assortment. Additionally, sequence analysis of the *RBT1* alleles in 18 representative clinical isolates from different clades showed hot spots of either deletion or insertion in the “PES/TSA/V” repetitions (repetition 2 in Figure 1A): deletions of 9 to 19 amino acids at position 377 of the full length protein or insertion of 28 amino acids at position 480 (data not shown). Finally, the strains harboring *RBT1* length polymorphisms were contained within a single clade suggesting an early origin of these modifications within the *C. albicans* genus (data not shown).

### Localization of the Rbt1 protein

**Figure 2:**
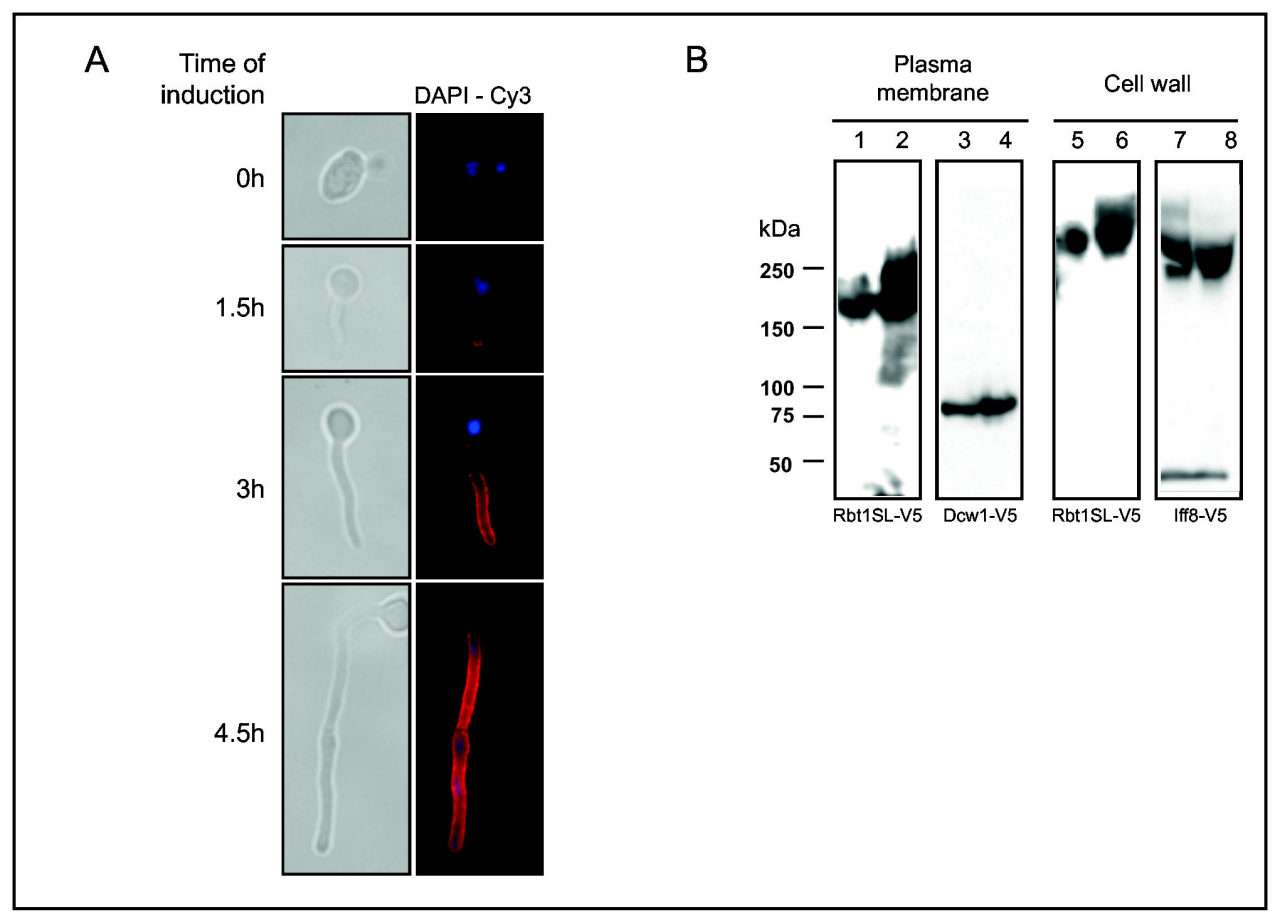
*In* *vivo* localization of Rbt1. A/ The V5-tagged Rbt1 expressed under the control of the *RBT1* promoter (VIF210) was detected by immunofluorescence after three different times of hypha induction. Fixed cells were directly treated first with anti-V5 antibodies and then with anti-mouse IgG-Cy3 coupled antibody, and immunofluorescence was observed using an Olympus BX51 microscope. B/ Western blot analysis of proteins solubilized either from the plasma membrane fraction (lanes 1 to 4) or from the cell walls (lanes 5 to 8) of yeast cells (odd numbers) or hyphae (even numbers): lanes 1, 2, 5 and 6, the strain VIF211 expressing the *RBT1SL-*V5 allele under the control of the *ACT1* promoter (*ACTIp*); lanes 3 and 4 the strain VIF106 expressing *DCW1*-V5 under *ACT1p* and lanes 7 and 8, the strain VIF105 expressing *IFF8*-V5 under *ACT1p*. In lanes 1 to 4, proteins from a membrane-enriched pellet (C_10000g_) were solubilized in the presence of 2% SDS; in lanes 5 to 8, the cell wall fraction (C_1000g_) was incubated in NaAc buffer + 2U of β-1,6-glucanase for 3 hours at 37°C to solubilize GPI-anchored cell wall proteins. Samples were separated by SDS-PAGE, transferred onto nitrocellulose membrane and immunoblotted with monoclonal anti-V5 antibodies.

In order to localize the Rbt1 protein in living cells, an epitope-tagged copy of each of the two alleles was constructed by insertion of a V5 sequence between amino acids 273 and 274, *ie.* just upstream of domain II (see Experimental procedures). These copies were targeted to the *RBT1* locus to express the tagged proteins under the control of the native *RBT1* promoter (VIF209 and 210, see Table 1). Since *RBT1* expression has been shown to be controlled by Tup1 [7], we perfomed immunofluorescence after 3 different times of hypha induction. Data in Figure 2A clearly indicated that Rbt1 was not present at the cell surface of yeast cells including those from which the hypha emerged but was present at the surface of the germ tube and progressively covered the surface of the entire hypha. The same pattern was observed for the two alleles (data not shown). This experiment confirmed that Rbt1 was exclusively exposed on the cell surface of hyphae.

Rbt1 is predicted to be GPI-modified and considering the two amino acid residues upstream of the putative ω site (F, E), the protein should be cell wall anchored [44]. To test this prediction, the V5-tagged RBT1FL and RBT1SL alleles expressed under the control of the *ACT1* promoter were targeted to the RPS1 locus as previously described (VIF211 and 212, see Experimental procedures and Table 1) [36]. Tagged strains were grown as yeast or hyphae before cell fractionation and western blotting. Cells expressing GPI-anchored V5-tagged Dcw1 protein and V5-tagged Iff8 protein were included as controls respectively of plasma membrane localization and cell wall localization [36]. As shown in Figure 2B, Rbt1 was detected in the β-1,6-glucanase solubilized cell wall fraction (lanes 5 and 6) as was the Iff8 control protein (lanes 7 and 8). However, Rbt1 was also present in the membrane-enriched pellet after solubilization with SDS (lanes 1 and 2) as observed for the Dcw1 control protein (lanes 3 and 4). This dual localization was observed for the Rbt1FL and Rbt1SL proteins independently of the cell morphology, even if slightly less protein was detected in the yeast form in comparison with hyphae. 

### Role of Rbt1 in adhesion to polystyrene

**Figure 3:**
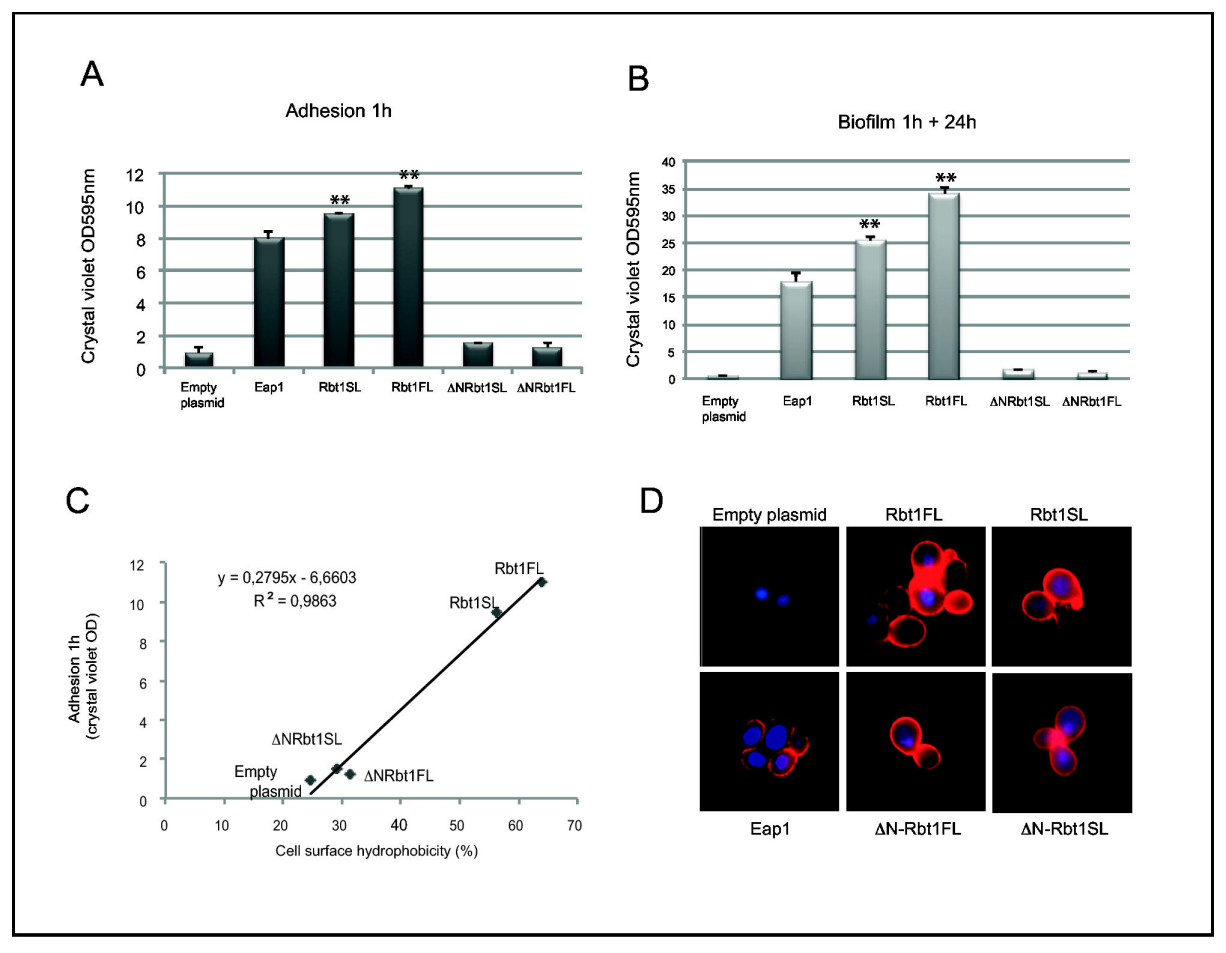
Adhesion and biofilm formation on polystyrene plates treated for cell culture by *S. cerevisiae* cells expressing entire or truncated Rbt1 variants. *S. cerevisiae* cells expressing Rbt1SL, Rbt1FL, ΔNRbt1SL and ΔNRbt1FL together with the control protein Eap1 and the pBC542 vector alone were tested for adhesion, biofilm formation and surface hydrophobicity (VIF201 to 206). A/ Cells were incubated in polystyrene plates for 1 h at 28°C, non-adherent cells were removed, and total biomass was measured immediately by crystal violet staining. B/ After additional 24h of incubation at 28°C in minimal medium the same measurement were done. For experiments A and B, the given values represent mean ± standard deviation of results of one experiment performed in duplicate and representative of three independent experiments. Pairwise comparisons were made by two-tailed Student’s T-test: significant comparison (p value<0.01) are indicated with two asterisks. C/ Cell surface hydrophobicity of *S. cerevisiae* cells expressing entire or truncated Rbt1 variants. Affinity to decane of *S. cerevisiae* recombinant strains was determined using the MATS method and the percentage of hydrophobicity was calculated for cells expressing the Rbt1SL, Rbt1FL, ΔNRbt1SL and ΔNRbt1FL proteins together with the control protein Eap1 and the pBC542 vector alone. A correlation curve between adhesion to polystyrene and cell surface hydrophobicity for the four Rbt1 constructs and the negative control was calculated using adhesion and hydrophobicity measures. D/ Cell surface detection of *S. cerevisiae* cells expressing Rbt1SL, Rbt1FL, ΔNRbt1SL and ΔNRbt1FL together with the control protein Eap1 and the pBC542 vector alone. Fixed cells were incubated first with anti-HA antibodies and then with anti-rabbit IgG-Cy3 coupled antibodies, and immunofluorescence was observed using an Olympus BX51 microscope.

In order to further characterize Rbt1 and assign functions to its different domains, we tested the adhesion properties conferred by different subdomains of Rbt1 using the S. *cerevisiae* surface display system [38]. The *RBTISL* and *RBT1FL* alleles deleted for the coding regions for the 27 C-terminal amino acid residues predicted to act as the GPI anchor addition signal, and truncated versions of *RBTISL* and *RBT1FL* encoding proteins lacking amino acid 21 to 272 (including domain I, see Figure 1) were cloned in the pBC542 vector. This plasmid allows the production of a fusion protein between the tested polypeptide, a HA tag, the *Candida glabrata* Epa1 Ser/Thr-rich region and the S. *cerevisiae* Cwp2 cell wall targeting signal [38]. Heterologous expression at the S. *cerevisiae* cell surface of the entire or the truncated Rbt1 proteins as well as the Eap1 adhesin was confirmed by immunofluorescence (Figure 3D). Analysis by flow cytometry of the four strains expressing Rbt1FL and Rbt1SL variants showed an equivalent level of protein at the cell surface (Table 3).

**Table 3:** FCM data on *S. cerevisiae* surface display strains.

**Strains**	**% fluorescent cells**	**Mean**
Empty plasmid (VIF205)	11%	5.4
Eap1 (VIF206)	44%	62.9
Rbt1SL (VIF201)	66%	51.1
Rbt1FL (VIF202)	60%	60.9
ΔNRbt1SL (VIF203)	74%	51.3
ΔNRbt1FL (VIF204)	57%	48.7

The adhesion properties on polystyrene of each of these strains were investigated. *S. cerevisiae* cells expressing each of the Rbt1 constructs together with an Eap1 positive control and cells containing the empty vector were incubated in 24-well tissue culture plates for 1 h at 28°C. Non-adherent cells were removed and the biomass was estimated by crystal violet staining. As shown in Figure 3A, after 1 hour of incubation, we observed that expression of the Rbt1FL and Rbt1SL proteins significantly enhanced *S. cerevisiae* adhesion to polystyrene (Student’s T-test, p<0.01). Indeed, the biomass levels were significantly higher: 10.6-fold and 12.4-fold higher for yeasts expressing the Rbt1SL and Rbt1FL fusion constructions, respectively, than the biomass observed for the control strain containing the empty vector. In the same experiment, the biomass level of the strain expressing the Eap1 fusion was 9-fold higher. Moreover, the S. *cerevisiae* recombinant strains that expressed the C-terminal domains of Rbt1FL or Rbt1SL displayed biomass levels similar to those measured for the strain carrying the empty vector, suggesting that the N-terminal domain of the Rbt1 protein was essential to mediate Rbt1-dependent adherence of *S. cerevisiae* to polystyrene either directly or because its absence modifies the protein in such way that the domain involved in adherence is no longer functional (Figure 3A). 

In parallel experiments, the initial attachment was followed by a 24h-incubation and biomass levels (after two washes) were again measured to examine the formation of biofilm. Figure 3B showed that the two forms of Rbt1 contributed significantly to *S. cerevisiae* biofilm formation (p<0.01): indeed, while the strain harbouring the empty plasmid was unable to form a biofilm and grew only as planctonic cells, a biofilm was observed for the recombinant strains after 24h. Additionally, the ratio of biomass accumulation to initial adhesion was 3.1 for the Rbt1FL expressing strain and 2.7 for Rbt1SL expressing strain suggesting that Rbt1 was both involved in cell-substrate and in cell-to-cell adhesion, and that these functions required the entire protein.

### Cell surface hydrophobicity of the *S. cerevisiae* recombinant strains

To characterize the cell surface properties of the strains expressing the different variants of Rbt1, we defined their cell surface hydrophobicity using a method based on decane affinity [39]. As shown in Figure 3C, the S. *cerevisiae* recombinant strains expressing Rbt1SL and Rbt1FL fusions had a percentage of cell surface hydrophobicity higher than that of yeasts containing the empty vector or expressing the ΔNRbt1SL or ΔNRbt1FL fusions. Notably, cell surface hydrophobicity of these strains was highly correlated with their ability to adhere to polystyrene (R^2^=0.986). Taken together, these data indicated that the adhesion property conferred by Rbt1 was mainly due to its hydrophobicity, a characteristic linked to its N-terminal domain even if as yet unknown characteristics of Rbt1 might play a role in these adhesion processes. 

### Adhesion and biofilm formation of two *C. albicans* *RBT1* overexpressing strains

In order to study the adhesiveness conferred by Rbt1 in *C. albicans* independently of other hypha-induced adhesins, we constructed *C. albicans* strains constitutively expressing high levels of Rbt1FL or Rbt1SL. For this purpose, the *RBT1* native promoter of either the *RBT1FL* or *RBT1SL* alleles was exchanged for the *TEF1* promoter, yielding strains OExRbt1FL and OExRbt1SL, respectively (VIF208 and 207, see Experimental procedures). Real time quantitative PCR analysis showed that in the yeast form *RBT1* mRNA levels reached 40.1% and 47.1% of those of *ACT1* mRNA for the *RBT1FL* and *RBT1SL* alleles, respectively, while *RBT1* mRNA represented less than 0.1% of *ACT1* mRNA in the wild type strain. In hyphae, *RBT1* mRNA levels increased to 47.2% and 61.4% of the *ACT1* mRNA levels for the *RBT1FL* and *RBT1SL* alleles, respectively. In the wild type context, the levels of *RBT1* mRNA represented 8.3% of those of *ACT1* mRNA. Thus strains OExRbt1FL (VIF208) and OExRbt1SL (VIF207) overexpressed *RBT1* in both yeast and hyphal forms. 

**Figure 4:**
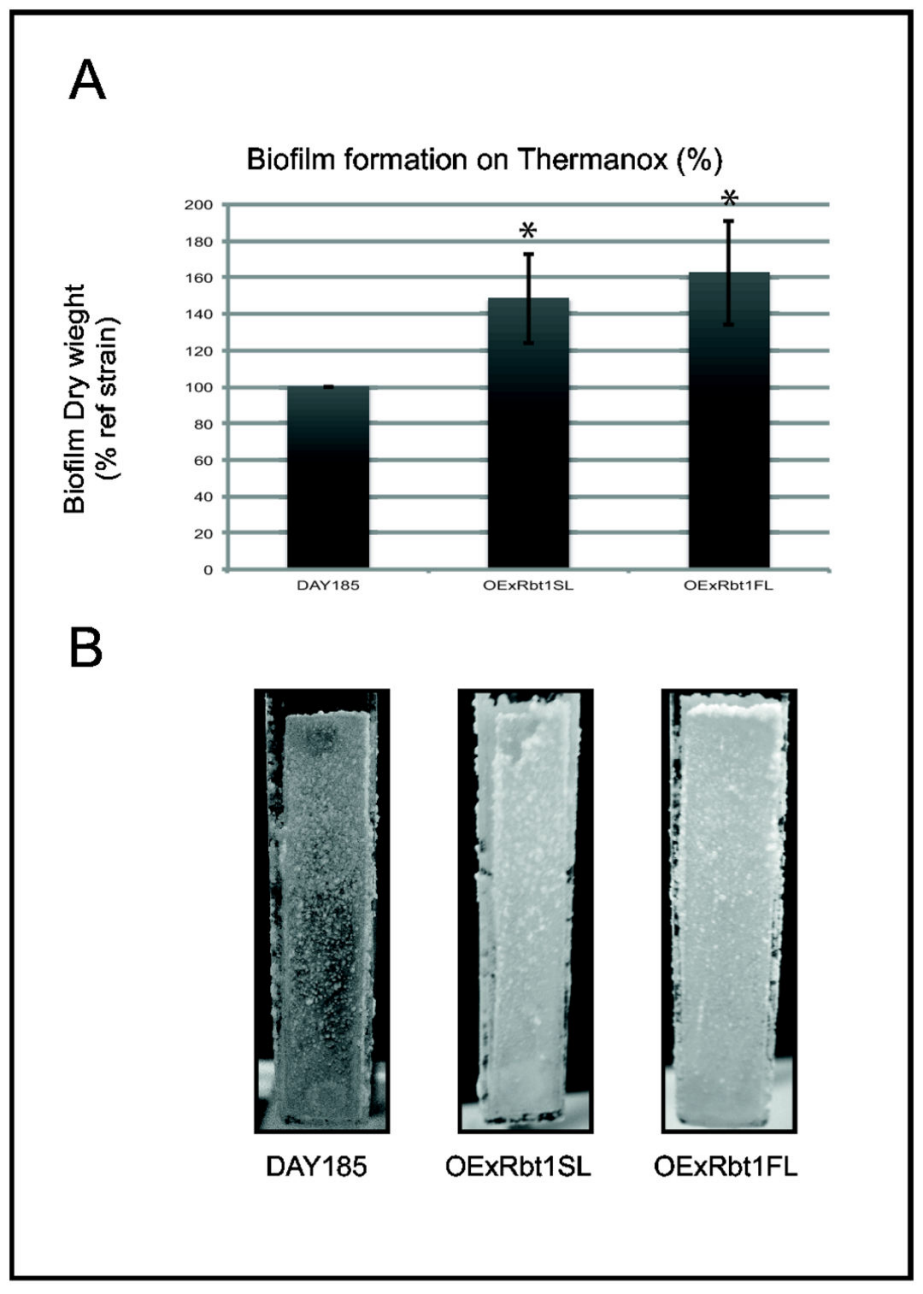
Biofilm formation on Thermanox™ in micro-fermenter of *C. albicans* strains overexpressing the Rbt1SL and Rbt1FL proteins (VIF 207 and 208). After an initial immersion period of 30 minutes in the inoculums, plastic slides were further incubated for 40 hours at 37°C in micro-fermenter. A/ Dried weight of each biofilm was measured. The percentages of biomass obtained for the two overexpressing strains were calculated in comparison to those of the wild type control strain which was fixed to 100%. Values given represent mean ± standard deviation (SD) of results of one experiment performed in duplicate and representative of three independent experiments. B/ Pictures of the three biofilms formed on the Thermanox™ lamella after 40 hours. Pairwise comparisons were made by two-tailed Student’s T-test: significant comparison (p value<0.05) are indicated with an asterisk.

Biofilm formation assays on Thermanox™ in micro-fermenters were performed for these two overexpressing strains as described by García-Sánchez et al. [35]. The results obtained in these conditions showed a thicker biofilm for the two overexpressing strains (Figure 4B) with the biomass measured for the OExRbt1SL and the OExRbt1FL strains after 40 hours of incubation being respectively 148% and 162% of that formed by the control strain, a difference statistically significant when compared to the reference strain (p<0.05) (Figure 4A). A greater biofilm formation of strain OExRbt1FL compared to OExRbt1SL was observed consistently over the different experiments but the difference was not statistically significant: this suggested that the Rbt1FL protein might have a higher capacity to promote biofilm formation than Rbt1SL. Microscopic observation of the Thermanox™ slides after the adhesion step did not reveal any difference between the three strains (data not shown).

### Adhesion of the two overexpressing strains to human cells

**Figure 5:**
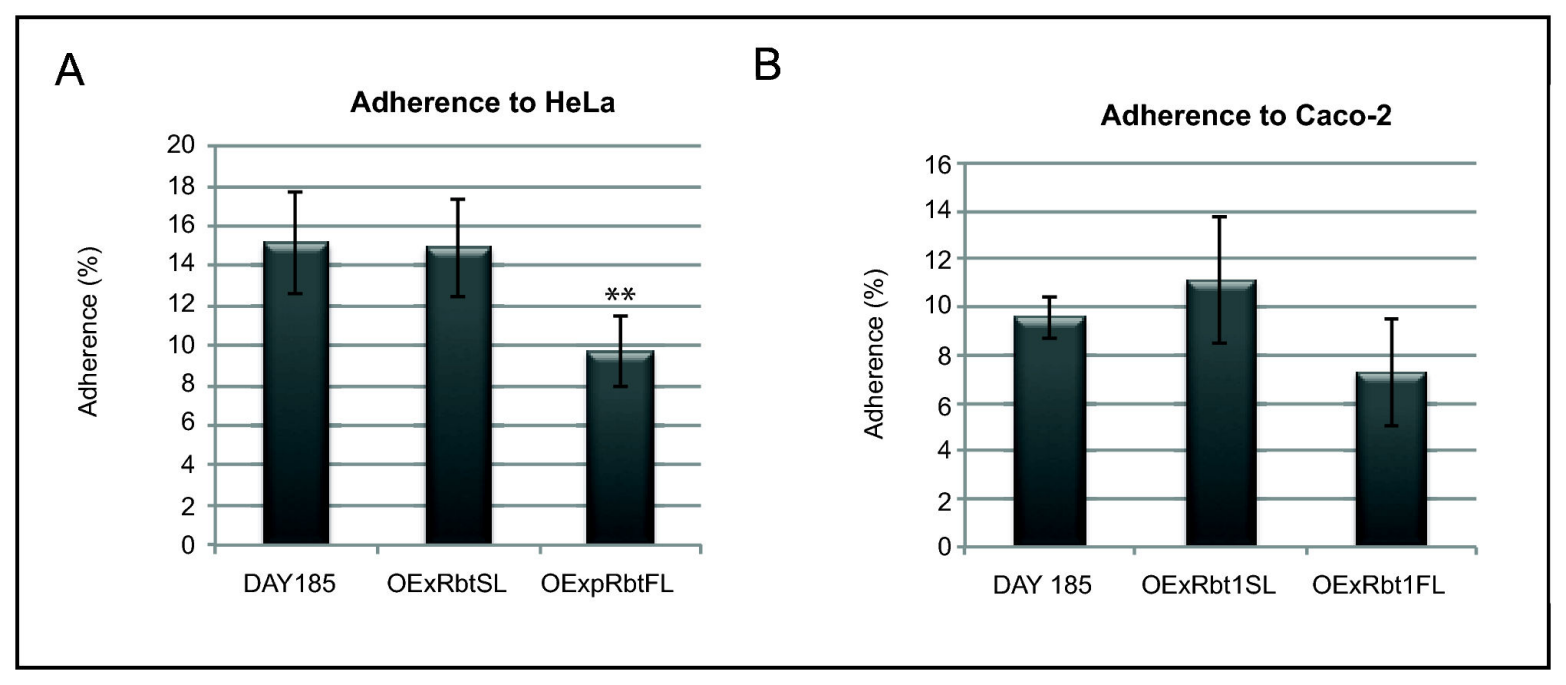
Adherence of *C. albicans* strains overexpressing the Rbt1SL and Rbt1FL proteins (VIF 207 and 208) to human cells. Yeasts cells were incubated with confluent HeLa cells for 45 minutes (A) or with Caco_2_ cells for 30 minutes (B). The percentage of adhesion represents the number of adherent yeasts reported to the number of yeasts in the inoculums. Values given represent mean ± standard deviation (SD) of results of one experiment performed in duplicate and representative of three independent experiments. Pairwise comparisons were made by two-tailed Student’s T-test: significant comparisons are indicated with two asterisks for p value<0.01.

We then tested the adherence to host cells of the two overexpressing strains using two models: human HeLa epithelial cells and human epithelial colorectal adenocarcinoma cells (Caco-2). As shown in Figure 5A, while adhesion to HeLa cells of the OExRbt1SL strain was similar to the adhesion of the control strain, the OExRbt1FL strain showed a significantly reduced percentage of adhesion in comparison with the two first strains (p <0,01). This result suggested that adherence of *C. albicans* was impaired by the expression of the longer *RBT1* allele in yeast cells. With Caco-2 cells again adherence of the OExRbT1FL strain showed a repeatable slight decrease in comparison with the control and the OExRbt1SL strains but not statistically significant (Figure 5B).

### Aggregation properties of the two overexpressing strains

**Figure 6:**
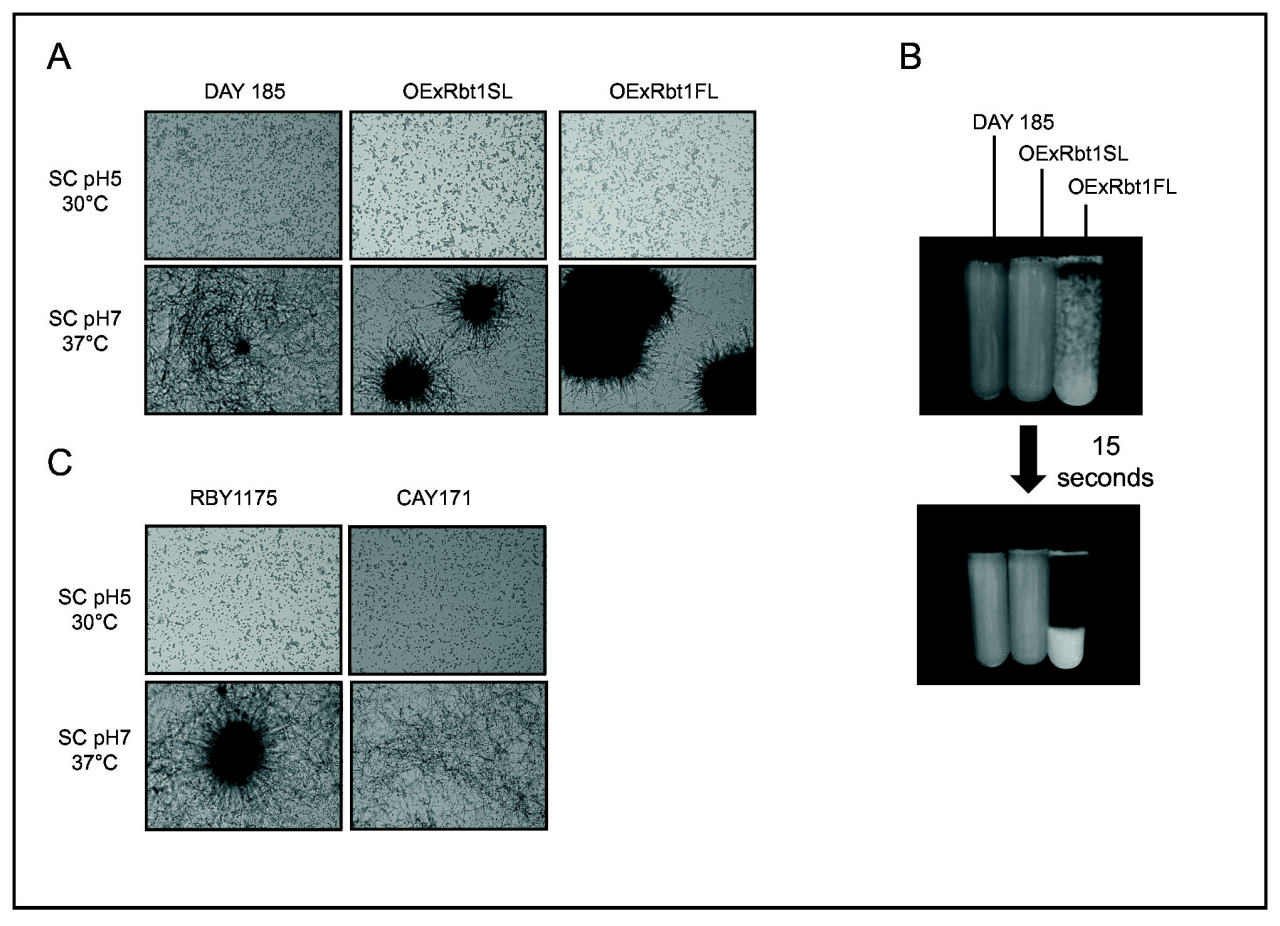
Aggregation assay of *C. albicans* strains overexpressing the Rbt1SL and Rbt1FL proteins (VIF 207 and 208) (A, B) or deleted for *RBT1* (CAY171) (C). Tested and control strains were cultivated either in unbuffered medium at 30°C or in pH7 buffered medium at 37°C for one night and examined by light microscopy (×40 magnification). Sedimentation of aggregates was allowed for the first set of strains and tubes were photographed after 15 seconds (B).

In a recent study, Ramsook et al. (2010) identified sequences with a high β-aggregation potential in Rbt1 using the prediction program TANGO (http://tango.crg.es/) [45]. Prediction for Rbt1 revealed the presence of two VTTGVVVVT sequences in Rbt1FL (at position 433 and 531, in domain III, see Figure 1) while only one was present in Rbt1SL at position 421. Thus we tested the ability of the two overexpressing strains to aggregate. Cells were grown overnight either in unbuffered SC medium at 30°C or in buffered SC medium (pH7) at 37°C in order to obtain cells in either the yeast or hypha form. As illustrated in Figure 6A, while no aggregates were detected when cells were in the yeast form, hyphae were able to form aggregates. Moreover, the OExRbt1FL strain (VIF208) formed much bigger aggregates than did the OExRbt1SL strain (VIF207). As a consequence of the large aggregates formed, the OExRbt1FL strain flocculated more quickly than the two other strains as shown in Figure 6B. To confirm the role of Rbt1 in hyphae aggregation a previously constructed *rbt1-/-* strain [19] was used in the same aggregation assay. As expected, formation of aggregates was abolished in the *rbt1-/-* strain (CAY171) in comparison with the control strain as presented in Figure 6C. We also confirmed, as published in Ene & Bennett’s study [19] that this strain was impaired in biofilm formation using polystyrene plates treated for cell culture (data not shown).

**Figure 7:**
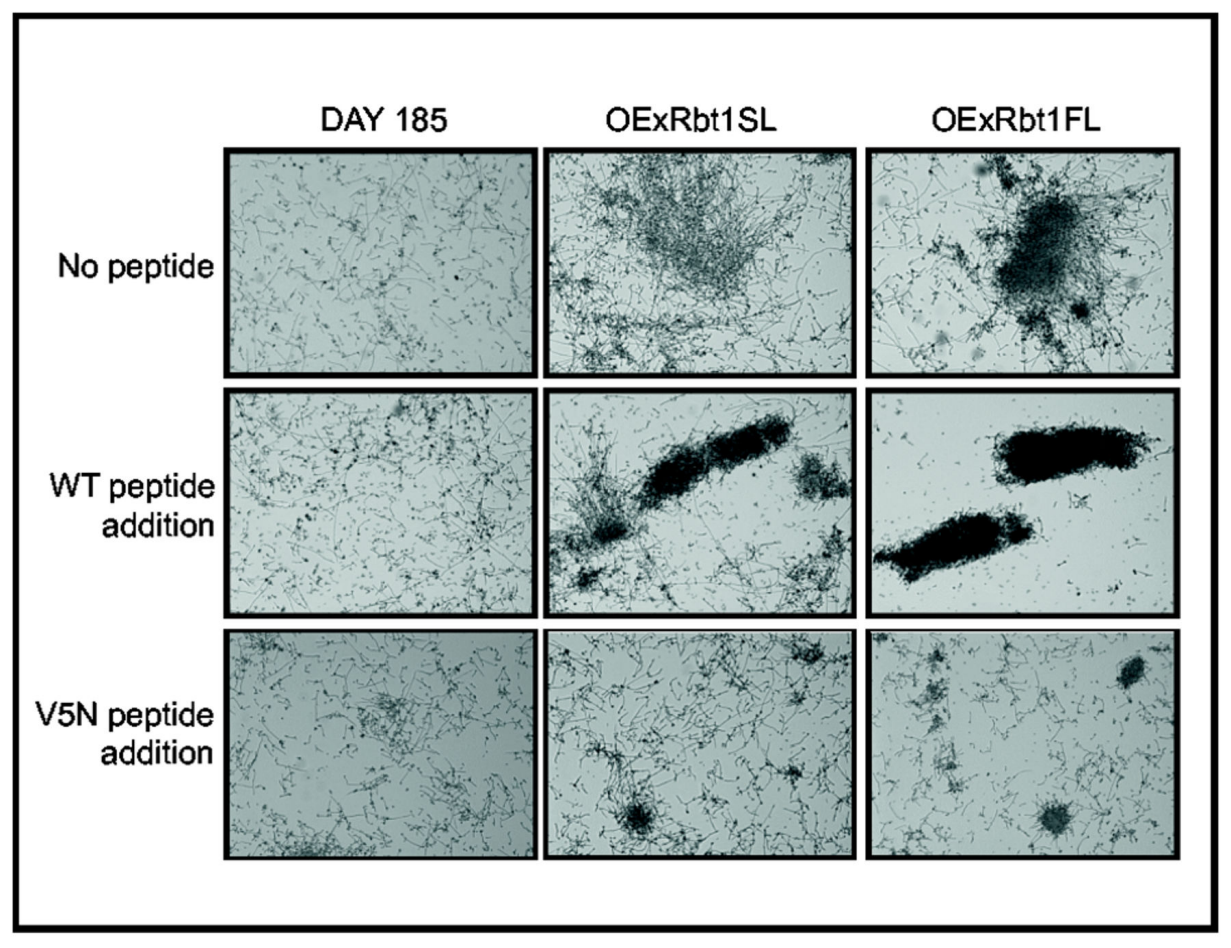
Aggregation stimulation and aggregation inhibition in the presence of wild type (VTTGVVVVT) and mutated (VTTGNVVVT) peptides added respectively at 2μg.mL^-1^ and 20μg.mL^-1^. After filamentation induction by pH and temperature switch (30°C pH5 to 37°C pH7) during 2 hours the strains were further incubated for 24 hours in the presence of the wild type high β-aggregation potential peptide or a mutated peptide. Cells were then examined by light microscopy (×40 magnification).

To confirm that the sequences with high β-aggregation propensity were directly involved in this phenomenon, we set up an experiment using two different peptides: (i) a wild-type peptide corresponding to the sequence found in both alleles; (ii) and a mutated peptide in which the valine at position 5 was replaced by an asparagine residue (V5N, VTTGNVVVT). The TANGO predictor no longer detected any β-aggregation potential for this mutated peptide. Filamentation was induced by growing cells in buffered medium for 2 hours at 37°C and cultures were stored for 24 hours at room temperature. Figure 7 clearly shows that the presence of the wild-type peptide enhanced aggregation for the two overexpressing strains in comparison with the culture without any peptide. On the contrary, incubation in the presence of the V5N peptide inhibited aggregation, demonstrating that the VTTGVVVVT sequence triggered the formation of aggregates and was critical for cell-to-cell association when *C. albicans* was in the hyphal form.

### Morphology-dependent cell surface exposure

**Figure 8:**
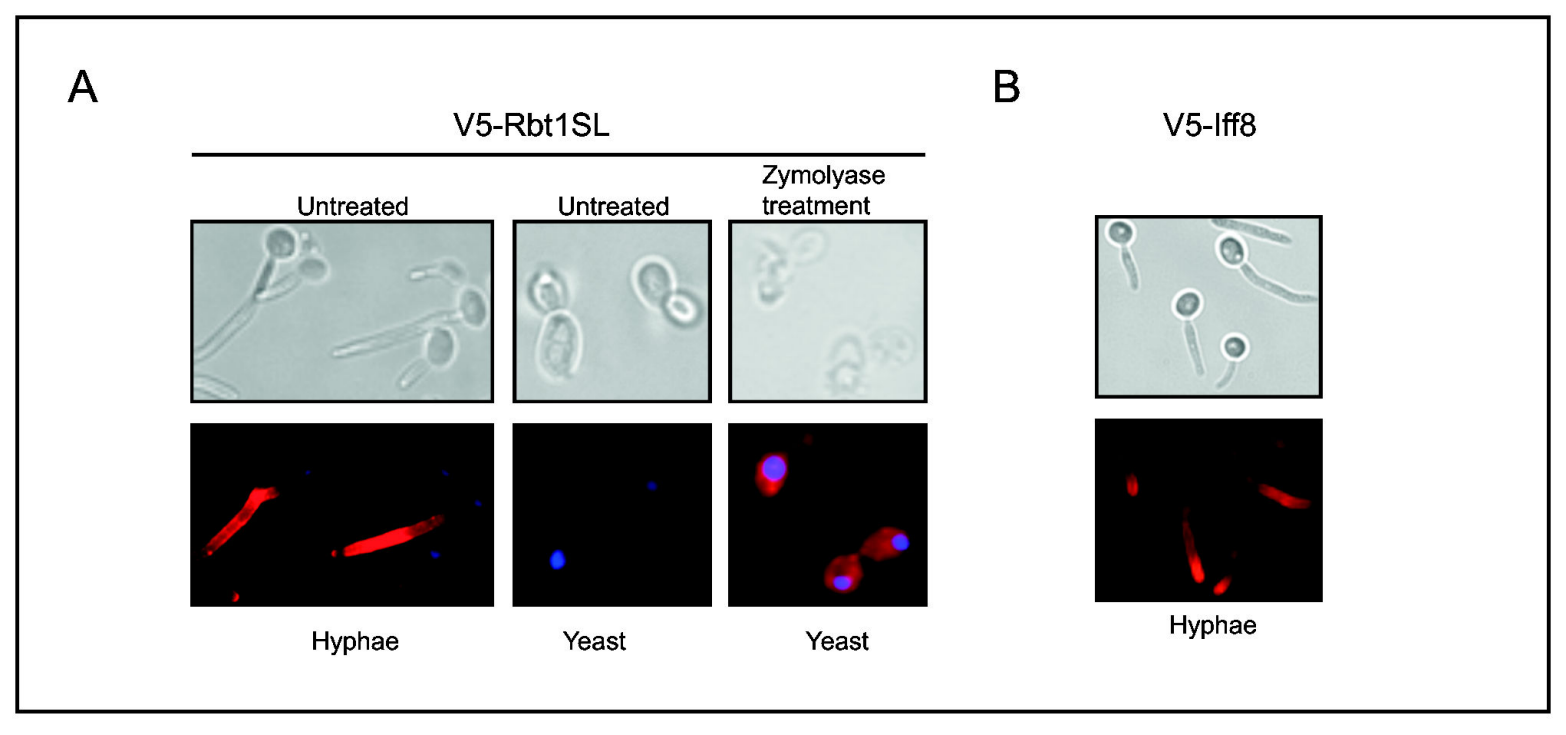
*In* *vivo* localization of Rbt1. A/ Cell surface detection of the *RPS1*-targeted *RBT1SL-V5* allele under the control of the *ACT1* promoter (VIF211) in untreated hyphae (left), untreated yeast (middle) and yeast after cell permeabilization (right). B/ Cell surface exposure of the *RPS1*-targeted *IFF8-V5* allele in hyphae. Fixed cells were incubated first with anti-V5 antibodies and then with anti-mouse IgG-Cy3 coupled antibody, and immunofluorescence was observed using an Olympus BX51 microscope.

The absence of any aggregation phenotype when cells were in the yeast form was unexpected since *RBT1* mRNA levels under the control of the *TEF1* promoter were shown to reach a high level in both forms. This result suggested that Rbt1 could not play its role in aggregation when cells were in the yeast form for a yet unknown reason. Two hypotheses could be anticipated: (i) its surface exposure differed between the two forms because Rbt1 was shown to be also present in the yeast form in strains expressing a V5-tagged Rbt1 protein (see above); (ii) Rbt1 needed a hyphal-specific protein partner to allow cell aggregation. To test the first hypothesis, an immunofluorescence experiment was performed on cells that expressed the V5-tagged Rbt1 protein under the control of the *ACT1* promoter grown either as yeast or as hyphae. The images presented in Figure 8A (left panel) showed that Rbt1 decorated the hyphal cell surface as observed for hyphae of a *C. albicans* strain expressing the V5-tagged Rbt1 protein from its own promoter (see Figure 2A). However, no signal was detectable on intact yeast cells (Figure 8A, middle panel) as also observed when the V5-tagged Rbt1 protein was expressed from its own promoter (Figure 2A), suggesting that the V5 epitope was not accessible to the antibody from the surface. Consequently, a permeabilization step was performed using zymolyase prior to the incubation with anti-V5 antibodies. After this treatment, yeasts that expressed a V5-tagged Rbt1 protein under the control of the *ACT1* promoter showed labeling (Figure 8A, right panel) while no signal appeared in yeast cells expressing the V5-tagged Rbt1 protein under control of its own promoter even after zymolyase permeabilization (data not shown). Consequently, although the V5-Rbt1 protein was present in the cell wall of both yeast and hyphae as shown by western blot (see *Localization of the Rbt1 protein*), the tag was specifically masked in the yeast form, suggesting a difference in the cell wall between the two cell morphologies.

In a previous study on the localization of proteins of the Iff family (GPI-anchored proteins), we performed immunofluorescence assays only on yeast cells [36]. In these conditions the cell wall protein Iff8 was undetected unless the cell wall was permeabilized with zymolyase, as Rbt1 in Figure 8A (right panel). Consequently we tested whether the change in cell wall structure in hyphae hypothesized in the case of Rbt1 could be confirmed with Iff8. Figure 8B shows that Iff8 could easily be detected without zymolyase treatment when cells were in the hyphal form confirming the data obtained with Rbt1.

## Discussion

 Much attention has been devoted to understand how *C. albicans* adheres to different substrates and to itself. These adhesion processes are considered of major importance in the first steps of colonization and biofilm formation, both being crucial phases occurring during *C. albicans* infection. To date many surface proteins have been implicated in the adhesion process such as the ALS family members, Hwp1 and Eap1 [46]. Hwp1 has two orthologues Hwp2/Pga8 and Rbt1; they share similarities mostly at the C-terminal domain and they all have been described as being involved in adhesion to different substrates. Interestingly they diverge in their N-terminal domain which is the domain specifically identified in Hwp1 as being a substrate for host transglutaminase activity, making this protein essential for attachment to buccal epithelial cells [18]. Therefore, we have investigated the contributions of the different domains of Rbt1 in adhesion and biofilm formation.

First we have studied Rbt1 surface localization, fundamental for a proposed role in adhesion: Rbt1 possesses a GPI anchor sequence signal at its C-terminus, like the two other members of the family Hwp1 and Hwp2 [17]. It is commonly accepted that GPI anchoring predictions are not 100% accurate due to problems in defining an exact C-terminus consensus for GPI-anchored proteins (GpiPs) [47]. Experimental demonstration is thus required to confirm localization to the cell envelope but also to demonstrate cell surface exposure, which is not seen for all GpiPs [36]. Our data indicated that Rbt1 is first deposited at the tip of the germ tube and subsequently distributed along the hyphae. In a previous study, trypsin treatment of live *C. albicans* yeast cells and hyphae suggested exposure of Rbt1 at the hyphal surface [48]. Our data are consistent with this observation and, furthermore, indicate the absence of Rbt1 at the surface of yeast cells. Moreover, we showed anchorage of Rbt1 both in the plasma membrane and the cell wall. While this dual location may be due to the artificially high levels of the V5-tagged Rbt1 protein that were necessary for detection by western blot, such problems have not been encountered when similar overexpression constructs were used to study the anchorage of proteins of the Iff family [36]. Therefore, the Rbt1 localization signal may be rather inefficient or the Rbt1 protein might be inefficiently transferred to the cell wall. Altogether our data show that: (i) Rbt1 is partially bound to the cell wall through bonds with β-1,6-glucans; (ii) Rbt1 is cell surface exposed and easily accessible on the exterior of the hyphae. Since Rbt1 has a GPI anchor signal these results suggest that this is a cell wall-GpiP although this has not been biochemically proven.

To decipher the role of the different Rbt1 domains, we expressed these domains in the surrogate host *S. cerevisiae* and monitored the effects on adhesion. This confirmed the data of Nobbs and co-workers [13], showing adhesion of the S. *cerevisiae* cells expressing the entire Rbt1. On the other hand, Rbt1 deleted of its N-terminus, although detected at the S. *cerevisiae* cell surface, was unable to promote adhesion of the recombinant yeasts to polystyrene, confirming that the N-terminal domain was necessary but not necessary sufficient for adhesiveness of Rbt1 to polystyrene. Determination of the surface properties of these strains proved that only *S. cerevisiae* recombinant cells expressing the entire Rbt1 showed an increased cell surface hydrophobicity therefore correlating surface hydrophobicity to adhesiveness. Interestingly when Rbt1 was overexpressed in *C. albicans* we observed an increase in hydrophobicity but the changes in adhesion were not as strong as in *S. cerevisiae* (data not shown). Taken together, these results implied that high level expression of a cell surface protein might modify the cell surface physico-chemical properties such as its hydrophobicity but that in *C. albicans* other parameters greatly influence the adhesiveness of the cell. These parameters might include the occurrence of other adhesins or the difference in composition and structure of the cell wall.

Rbt1 possesses a C-terminus containing two 42 amino acid-long motifs shared by previously characterized *C. albicans* adhesins such as Hwp1 and Eap1 (see Figure 1). Yet, amplification of the *C. albicans RBT1* coding sequence in BWP17 produced two alleles, namely *RBT1FL* and *RBT1SL*, with a 420 nucleotides-difference in size. Interestingly Rbt1SL contrary to Rbt1FL possesses only one 42 aa-long motif. Characterization of the *RBT1* loci in different clinical isolates revealed allelic variations from one strain to another and within strains. However, none of the clinical isolates harbored the *RBT1SL* allele, suggesting that this allele recently emerged probably resulting from a recombination event between repeats within Rbt1. Here, we have taken advantage of the *RBT1FL* and *RBT1SL* alleles to understand the role of the Rbt1 42 amino acid-long motifs in adhesion and observed that the Rbt1FL protein exhibited a slight increase in capacity to promote biofilm formation, but it did not affect the initial adherence to polystyrene. These observations may reflect a role of the 42 aa-long motif in directly mediating cell-to-cell interactions during biofilm formation or in modifying the exposure of an interacting domain. Notably, a defect in biofilm formation has been observed by Padovan et al. [49] for *C. albicans* cells expressing a Hwp1 variant with a 34 amino acid-long deletion in a serine and threonine-enriched domain.

Recently, Garcia et al. [15] have uncovered amyloid-forming sequences in the Als5 protein that contribute to cell aggregation and biofilm formation. Since the Rbt1FL and Rbt1SL proteins differed by the number of repeats of a peptide with high β-aggregation potential (VTTGVVVVT), we hypothesized that strains overexpressing *RBT1FL* or *RBT1SL* would have different aggregation phenotypes. A liquid aggregation assay gave no result when cells were grown as yeasts but once filamentation was induced, we observed a massive aggregation of hyphae for the strain that expressed the full length Rbt1 protein in comparison with the wild type strain while the strain overexpressing the short length protein displayed an intermediate aggregation phenotype, strongly suggesting a role of the VTTGVVVVT sequences in aggregation. Garcia et al. [15] have shown that amyloid-dependent clustering of Als5 increases the avidity and the strength of adherence mediated through the N-terminal domain. A similar mechanism might operate in Rbt1 with the Rbt1 N-terminus acting as the “substrate-binding domain” and the putative aggregate-forming sequences inducing the clustering of different molecules of Rbt1 thereby increasing adhesiveness and cell-to-cell interaction. The difference of aggregation observed between yeast and hyphae could explain the observation of a more fragile biofilm in the micro-fermenter experiments. Indeed the biofilm structure is reported to be composed of a layer of yeast cells upon which a mixture of pseudo-hyphae and hyphae are forming the mature biofilm embedded in an extracellular matrix. In the case of Rbt1 overexpressing strains, the mature layer would have been weakly attached to the support due to fragile interactions with the layer of yeast cells but would have formed a compact layer easily washed off by the flow through.

Most of the adhesins characterized in *C. albicans* not only mediate adhesion to abiotic surfaces but also interaction with human cells. For instance Eap1 was shown to confer adhesion of recombinant *S. cerevisiae* to human HEK293 kidney epithelial cells [50] and Hwp1 was shown to be covalently cross-linked to mammalian epithelial cells [18]. Consequently, we tested whether Rbt1 could confer properties of adherence to host cells. Overexpression of Rbt1SL did not affect adhesion of *C. albicans* yeast cells to human cells. In contrast, overexpression of Rbt1FL reduced adhesion of yeast cells, suggesting that increased levels of this form of Rbt1 could either mask proteins necessary for *C. albicans* interaction with host cells (Hwp1 for example) or prevent them from interacting by forming intermolecular bonds. Indeed, according to the model of Ramsook et al. [14], Rbt1FL overexpression could trigger the clustering of other cell surface proteins harboring aggregate-forming sequences such as Hwp1 and Eap1 and thus interferes with the attachment to host cells.

Finally, our results have shown a striking difference in the cell surface accessibility of the V5-tagged Rbt1 protein in yeast and hyphal cells. Indeed, while this protein showed similar cell wall anchorage in the two cell types, it was accessible to anti-V5 antibodies only in hyphal cells. Similar observations have been made for the Iff8 protein. Indeed, we have previously shown that this 714 amino acid-long cell wall-anchored protein was not long enough to be cell surface-exposed in the yeast form [36]. That similar results were observed in the case of the V5-tagged Rbt1 protein was not unexpected since only 477 amino acid residues are found downstream of the V5 tag in this protein. Most interestingly, we could show that gentle enzymatic digestion of the yeast cell wall could uncover the V5 epitope harbored by the V5-tagged Rbt1 and Iff8 proteins, suggesting that these proteins are too deeply embedded in the yeast cell wall matrix to be reached by anti-V5 antibodies unless cell wall glucans are enzymatically degraded. Because the anchoring in the cell wall of these proteins does not differ according to *C. albicans* morphology, the difference in accessibility of these cell wall proteins in yeast and hyphal cells is likely to result from changes in the cell wall organization between these morphological types. Notably, Cheng et al. [51] have described a reduction in the cell wall fimbriae layer in hypha: the average fibril length decreased from 0.116 µm in yeasts to 0.073 µm in hyphae, thus influencing detection by dectin-1. Wheeler and collaborators showed that glucans were more accessible to antibodies in the hyphae than in the yeast form; confirming also that the accessibility of cell wall compounds was very different between the two cell forms [52]. Therefore, major changes in the structure of the cell wall, possibly at the level of the fimbriae layer, might allow shielding of cell wall proteins in yeast cells and their unmasking in hyphal cells. This suggests an additional, unexpected layer of regulation for the function of cell wall proteins in addition to transcription regulation and post-translational modification. This new form of regulation may provide *C. albicans* with an additional means to control its surface qualities and properties.
